# Antifungal susceptibility of non-albicans *Candida* spp. isolated from raw milk and human blood in Alborz and Tehran provinces

**Published:** 2019-12

**Authors:** Zahra Namvar, Abbas Akhavan Sepahy, Robab Rafiei Tabatabaei, Sassan Rezaie

**Affiliations:** 1 Depatment of Microbiology, School of Biological Sciences, Islamic Azad University, North Tehran Branch, Tehran, Iran; 2 Depatment of Medical Mycology and Parasitology, Division of Molecular Biology, School of Public Health, Tehran University of Medical Sciences, Tehran, Iran

**Keywords:** *Candida parapsilosis*, *Candida glabrata*, *Candida krusei*, *Candida tropicalis*, Antifungal, Raw milk

## Abstract

**Background and Objectives:**

Recent reports indicate high prevalence of fungal infections due to non-albicans *Candida* spp. which are present in various environments such as raw milk. The quality of milk for fungal normal flora was investigated in this study.

**Materials and Methods:**

A total of 262 milk samples were collected directly from milk collection tanks indesignated dairy farms and cultured in SDA media. By further analysis of grown yeasts, 69 non-albicans *Candida* strains were identified. Antifungal susceptibility of the isolated species, were evaluated against amphotericin B, itraconazole, fluconazole and flucytosine. Fifty two non-albicans clinical samples isolated from human blood have been evaluated along.

**Results:**

Antifungal susceptibility evaluation in non-albicans strains isolated from milk revealed *Candida glabrata* and *Candida tropicalis* to be 100% sensitive to flucytosine and fluconazole. *Candida krusei* showed 94% and 80% sensitivity to flucytosine and fluconazole respectively. *Candida parapsilosis* indicated 72.72% sensitivity to fluconazole.

**Conclusion:**

Evaluation of non-albicans *Candida* species in raw milk and antifungal susceptibility patterns of these isolates-compare with non-albicansisolates from human blood, may help physicians to choose an appropriate medication for diseases needing long-term treatment, especially for diseases caused by local strains.

## INTRODUCTION

Fungi are eukaryotic microorganisms which can be present in different environments. Raw milk of livestock such as cows is considered; as a complete food source, and a proper environment for some fungi such as yeasts.

The secondary metabolites of fungi, such as mycotoxins, are generally of public health importance and have been studied excessively in milk. The prevalence of fungi in raw milk as well as the drug resistance of fungi associated with raw milk normal flora is worthy of consideration in terms of public health (1).

*Candida* are small, thin-walled yeasts that propagate through budding. Candidiasis is an infection caused by different species of candida, especially *C. albicans*. In the last two decades, the frequency and severity of infections have increased dramatically following the widespread use of antibiotics, steroids and other immunosuppressive drugs. *Candida* species are found everywhere in human and animal coexistence. Numerous species cause candidiasis, in different parts of the body. Deep-systemic candidiasis involves blood or other internal organs are seen in people with weak or defective immune systems (2–4).

Recently, a high prevalence of nosocomial infections of yeast origin has been reported in which *C. albicans* is mainly implicated (80%) (5). However, non-albicans *Candida* spp. are the main opportunistic pathogens associated with comorbidity in patients with immunodeficiencies, cancers, and organ transplant recipients (6). The most important challenge in the facing infections caused by non-albicans candida spp is their drug resistance to conventional antifungal drugs. These species, known as emerging pathogens, are the cause of uncontrolled nosocomial infections.

In this study, we investigated the presence of non-albicans *Candida* spp. in raw cow milk and evaluated their antifungal resistance profile compare to clinical isilates of non-albicans *Candida* spp. The results may bring us better knowledge about the yeast content in raw cow-milks and related antifungal resistance profile.

## MATERIALS AND METHODS

### Sample preparation.

Sampling was carried out during the four seasons of spring, summer, autumn, and winter. A total of 262 milk samples were taken from 14 designated dairy farms located in Tehran and Alborz provinces. Using sterile Falcon tubes, about 10 ml of milk were taken from raw milk-collecting tanks in the mentioned farms and were transported to the laboratory for further processing.

### Isolation of strains.

Milk samples were cultured on Sabouraud dextrose agar (SDA) culture media containing penicillin and streptomycin and were incubated at 37°C for one week. Obtained yeast colonies were then examined using candida chrom-agar culture media and were primarily differentiated based on their colors. However, for complete identification and confirmation, a part of rDNA in the ITS region has been amplified for sequencing in all isolates. Briefly, high molecular weight DNA was extracted from the samples using the glass bead and phenol-chloroform method as described previously (7). Using specific nucleotide primers for ITS region; ITS1-S:3′TCC GTA GGT GAA CCT GCG G5′ and ITS4-AS:3′ TCC TCC GCT TAT TGA TAT GC5′, a part of 28S of rDNA was amplified in each isolate by PCR method as described previously (8). Nucleotide sequencing of the obtained amplicons were performed and the results were compared in gene data bank (NCBI, NIH) for confirmation of identity.

### MIC analysis.

Antifungal susceptibility testing was performed for obtained non albicans isolates including: *C. parapsilosis, C. krusei, C. glabrata* and *C. tropicalis* against amphotericin B, itraconazole, fluconazole and flucytosine. Minimum inhibitory concentration (MIC) for the growth of these yeasts was determined using clinical and laboratory standard protocol (CLSI, M27-A3) and microdilution method (9). Fungi were grown on SDA culture media at 35°C for 48 h. About 1mm of grown yeast colonies were dissolved in 7 ml of sterile distilled water. The cell density of the resultant suspension was adjusted at a wavelength of 530 nm by adding enough sterile water to obtain 75–77% transmittance which indicated cell density as 1–5 × 10^6^ cells/m in this suspension. The initial suspension was then diluted with RPMI 1640 broth medium to achieve a working suspension with cell density of 0.5–2.5 × 10^3^ cells/ml.

In this study, *C. parapsilosis* ATCC 22019 was used for quality control.

## RESULTS

### Identification of samples.

Different types of yeasts were isolated and identified from 66% of 262 milk samples. Due to their similarity to common pathogens in clinical sapmles, the antifungal resistance test was performed on 4 species including; *C. tropicalis, C. parapsilosis, C. glabrata, C. krusei.* Identified *C. krusei* and *C. parapsilosis* samples were isolated at all seasons, while *C. tropicalis* was not isolated in winter and *C. glabrata* was isolated only in the winter.

At whole 69 non-albicans candida strains were isolated in 262 collected milk samples. Based on ITS sequencing results, 2 cases (0.76%) were identified as *C. glabrata*, 6 cases (2.29%) as *C. tropicalis*, 11 cases (4.19%) as *C. parapsilosis* and 50 cases (19.8%) as *C. krusei.*

### MIC analysis.

The results of MIC analysis for raw milk samples showed the MIC_50_ value offlucytosine as 0.031, 0.125, 0.062 and 1 for *C. glabrata, C. parapsilosis, C. tropicalis* and *C. krusei* strains respectively. MIC analysis for human blood samples showed that the MIC_50_ value of flucytosine was 0.062, 0.125, 0.062 and 1 for *C. glabrata, C. parapsilosis, C. tropicalis* and *C. krusei* strains. The results of MIC analysis showed that MIC_50_ of flucytosine was more effective on all tested specimens except for *C. krusei*. For *C. krusei* strains, itraconazole with MIC_50_ = 0.5 was more effective than flucytosine with MIC_50_ = 1. Besides, the drug of choice for *C. glabrata, C. parapsilosis* and *C. tropicalis* strains were also determined to be itraconazole with MIC_50_ = 1.125 (Table 1).

**Table 1.** Susceptibility yeast non-albicans *Candida* from raw milk tested against four antifungal

**Isolates (Number)**	**Antifungal Drugs**	**MIC Range**	**MIC (μg/mL)**		

			**MIC_50_**	**MIC_90_**	**GM**
*C. glabrata* (n=2)	fluconazole	1–16	8.5	-	4
	itraconazole	0.25–2	1.125	-	0.707107
	flucytosine	0.031	0.031	-	0.031
	amphotericin B	0.5–4	2.25	-	1.414214
*C. parapsilosis* (n=11)	fluconazole	0.125–32	0.5	28.8	1.208089
	itraconazole	0.015–8	0.25	8	0.497058
	flucytosine	0.031–0.125	0.125	0.45	0.096796
	amphotericin B	0.25–4	1	4	1.134313
*C. tropicalis* (n=6)	fluconazole	0.25–2	1	-	0.890899
	itraconazole	0.125–0.25	0.25	-	0.19708
	flucytosine	0.31–0.5	0.062	-	0.078325
	amphotericin B	0.25–4	1.5	-	1.259921
*C. krusei* (n=50)	fluconazole	0–64	4	64	3.238416
	itraconazole	0.0125–16	0.5	2	0.32989
	flucytosine	0–16	1	4	0.45007
	amphotericin B	0.0125–16	2	8	0.859421

Table 1 shows *in vitro* susceptibility of Non-albicans *Candida* yeast from raw milk tested against four antifungal. In the study, all isolates of *C. glabrata* and *C. tropicalis* as well as 94% of *C. krusei* isolates were sensitive to flucytosine. The sensitivity of *C. glabrata, C. tropicalis, C. parapsilosis* and *C. krusei* strains to fluconazole were 100%, 100%, 72.72% and 80% respectively. This finding showed the high effect of this antimicrobial medication on non-albicans *Candida* spp. The frequency of sensitive and resistant strains of non-albicans *Candida* isolated in raw cow-milk is shown in Table 2.

**Table 2.** Interpretation of the sensitivity results of *Candida* non-albicans isolates from raw milk evaluated based on CLSI-M27-A3 proposed breakpoints or ECV.

**Species (Number)**	**Antifungal Drugs**	**Sensitive N (%)**	**Dose-Dependent N (%)**	**Resistant N (%)**
*C. glabrata* (n=2)	fluconazole	2 (100)	-	-
	itraconazole	-	1 (50)	1 (50)
	flucytosine	2 (100)	-	-
	amphotericin B	1 (50)	-	1 (50)
*C. tropicalis* (n=6)	fluconazole	6 (100)	-	-
	itraconazole	2 (33.33)	4 (66.66)	-
	flucytosine	6 (100)	-	-
	amphotericin B	3 (50)	-	3 (50)
*C. parapsilosis* (n=11)	fluconazole	8 (72.72)	1 (9.09)	2 (18.18)
	itraconazole	4 (36.36)	2 (18.18)	5 (45.45)
	flucytosine	11 (100)	-	-
	amphotericin B	6 (54.54)	-	5 (45.45)
*C. krusei* (n=50)	fluconazole	40 (80)	4 (8)	6 (12)
	itraconazole	14 (28)	20 (40)	16 (32)
	flucytosine	47 (94)	3 (6) Intermediate	-
	amphotericin B	24 (48)	-	26 (52)

In this study, a comparison was done regarding the susceptibility testing results between the isolated cow milk and clinically isolated non-albicans *Candida* strains. It was shown that clinical non-albicans *Candida* isolates were also sensitive (100%) to flucytosine. MICs of antifungal medications and the frequency of resistant and sensitive clinical non-albicans *Candida* isolates used in this study have been shown in Tables 3 and 4, respectively.

**Table 3.** Susceptibility of non-albicans *Candida* from human tested against four antifungal.

**Isolates (Number)**	**Antifungal Drugs**	**MIC Range**	**MIC (μg/mL)**		

			**MIC_50_**	**MIC_90_**	**GM**
*C. glabrata* (n=24)	fluconazole	0.25–32	16	64	9.281035
	itraconazole	0.062–2	1	2	0.672757
	amphotericin B	0.25–1	1	1	0.707106
	flucytosine	0.5–0.031	0.062	0.5	0.085471
*C. parapsilosis* (n=17)	fluconazole	0.125–64	0.5	64	0.567889
	itraconazole	0.015–2	0.125	0.5	0.143093
	amphotericin B	0.06–1	0.5	1	0.431183
	flucytosine	0.5–0.031	0.125	0.5	0.111015
*C. tropicalis* (n=10)	fluconazole	4–64	16	64	24.870501
	itraconazole	1–16	8	16	7.511447
	amphotericin B	0.5–1	1	1	0.777203
	flucytosine	0.5–0.031	0.062	0.4625	0.071391
*C. krusei* (n= 1)	fluconazole	0.5–8	2	-	2.677899
	itraconazole	0.25–2	0.5	-	0.629960
	amphotericin B	0.25–4	1	-	1.122462
	flucytosine	1	1	-	1

**Table 4.** Interpretation of the sensitivity results of non-albicans *Candida* isolates from human evaluated based on CLSI-M27-A3 proposed breakpoints or ECV

**Species (Number)**	**Antifungal Drugs**	**Sensitive N (%)**	**Dose-Dependent N (%)**	**Resistant N (%)**
*C. glabrata* (n=24)	fluconazole	15 (62.5)	9 (37.5)	-
	itraconazole	1 (4.2)	8 (33.3)	15 (62.5)
	amphotericin B	24 (100)	-	-
	flucytosine	24 (100)	-	-
*C. parapsilosis (n=17)*	fluconazole	16 (94.1)	-	1 (5.9)
	itraconazole	10 (58.8)	6 (35.3)	1 (5.9)
	amphotericin B	17 (100)	-	-
	flucytosine	24 (100)	-	-
*C. tropicalis* (n=10)	fluconazole	-	1 (10)	9 (90)
	itraconazole	-	-	10 (100)
	amphotericin B	10 (100)	-	-
	flucytosine	24 (100)	-	-
*C. krusei* (n=1)	fluconazole	1 (100)	-	-
	itraconazole	-	-	1 (100)
	amphotericin B	1 (100)	-	-
	flucytosine	1 (100)	-	-

MIC ranges of fluconazole, itraconazole, amphotericin B, and flucytosine against the non-albicans *Candida* spp. in this study were 0–64, 0.015–16, 0.0125–16 and 0.031–16 μg/m respectively, while MIC ranges of fluconazole, itraconazole, amphotericin B, and flucytosine against the clinically isolated non-albicans *Candida* spp. were 0.125–64, 0.015–16, 0.06–4 and 0.031–1 μg/mL, respectively.

## DISCUSSION

Since milk is mentioned as a complete food source in all age groups particularly in children and in elderly, its quality and drug resistance rate of its microorganisms are of great importance because they may be substituted for the body natural flora.

In this study, from a total of 69 non-albicans *Candida* strains isolated from raw milk, 2 isolates (0.76%) were identified as *C. glabrata*; both of which were sensitive to flucytosine and fluconazole. One of these two isolates was resistant to itraconazole and sensitive to amphotericin B, and the other one was dose-dependent to itraconazole and resistant to amphotericin B.

Besides, 6 isolates (2.29%) were identified as *C. tropicalis*; all of which were sensitive to flucytosine and fluconazole. Of these, 2 cases were sensitive and 4 cases were dose-dependent to itraconazole. Among 6 isolates of *C. tropicalis*, 3 were sensitive and 3 were resistant to amphotericin B.

*C. tropicalis* species is considered as one of the common causes of disease in patients hospitalized in urinary and blood infections ward. Besides, *C. tropicalis* has become resistant to amphotericin B due to the long-term treatment by this antibiotic (10). According to recent studies, *C. tropicalis* use biofilm formation as a pathogenic agent, which may also be the cause of increased resistance to antifungal drugs (10).

In this study, 11 isolates (4.19%) were identified as *C. parapsilosis*; all of which were sensitive to flucytosine and fluconazole, except for 3 cases that were not sensitive to fluconazole. One of these 3 cases was dose-dependent, and the other two were resistant to fluconazole. In addition, among the 11 *C. parapsilosis* strains, 5 cases were resistant, 2 cases were dose-dependent, and the rest were sensitive to itraconazole. Regarding amphotericin B, 6 cases were sensitive, and 5 cases were resistant.

From the total of 69 non-albicans *Candida* strains, 50 (19.8%) were identified as *C. krusei*; all of which were sensitive to flucytosine, except for 3 cases which were reported as intermediate. Among the 50 isolates of *C. krusei*, 6 were resistant, 4 were dose-dependent, and 40 were sensitive to fluconazole. Also, 16, 20 and 14 isolates were resistant, dose-dependent, and sensitive to itraconazole respectively. Regarding amphotericin B, 26 isolates were resistant, and 24 isolates were reported as sensitive.

The most common cause of nosocomial infections among the *Candida* species is *C. albicans*, accounting for 80% of nosocomial infections, while *C. glabrata, C. parapsilosis* and *C. tropicalis* account for 50, 50 and 10–25% of these infections. On the other hand, the presence of factors causing drug resistance in these fungi has caused most studies to be done on these microorganisms (5). Non-albicans *Candida* resistance to azolic antifungal agents has made the treatment of infections caused by these microorganisms to be difficult; therefore, the high prevalence of infections with these yeast is a serious challenge (11–13).

Recent studies have shown that fungi isolated from livestock were azoles resistant, suggesting that they were independent of the host type. Resistant strains can also live in healthy individuals as a reservoir of resistant strains (12). It is worth noting that increased fungal infections due to the difficulty in diagnosing fungal pathogenic agents and their resistance to commercial antifungal medications have led to an increase in mortality and morbidity rate, especially in patients with immunodeficiency or cancer (14). Today, fungal species and genera which are less sensitive or resistant to antifungal agents are considered as the main causes of invasive infections in patients with immunodeficiency and are more under consideration (15).

The prevalence of these pathogenic agents sometimes increases the resistance to antifungal medications in theses fungi, leading to an increase in drug use, including azoles. Therefore, it is necessary to specify the MIC value of antifungal medications in the laboratory before starting the treatment (5, 16, 17).

There were similarities and differences between the results of the present study, examining non-albicans *Candida* spp. isolated from livestock, and the results of other studies, examining clinical samples. MIC values obtained in this study for *C. glabrata, C. parapsilosis* and *C. tropicalis* isolates were not consistent with MIC results of clinical study of Baghdadi et al. (2016). The MIC of amphotericin B against the *C. krusei* isolates was consistent with the MIC results of Baghdadi’s study, but no other MIC was consistent (7). This inconsistency was due to the higher sensitivity of milk-isolated strains to the azoles. These findings can be attributed to the non-use of antifungal medications in livestock and less exposure of these microorganisms to antifungal drugs. Today, the presence of some factors have caused various antifungal drugs to be identified, including the increase in fungal infections, changes in various fungi epidemiology, fungi resistance to antifungal drugs, and new toxins production by them (18). Azoles with MIC_50_ to MIC_90_ are commonly used for the treatment of infections caused by yeasts (19).

There were similarities and differences between the results of present study and those of Mendes et al. (2018) study which conducted on antimicrobial susceptibility of fungi strains isolated from clinical samples, wild animals, and cow’s mastitis. Mendes et al. (2018), indicated that among the non-albicans *Candida* spp. used in the present study. In their study, only *C. parapsylosis* was common and the rate of sensitive isolates to amphotericin B was higher than those reported in the present study, but their results for flucytosine, and fluconazole are similar (5).

The specimens identified in this study were in common with those of Du et al. (2018), however, different results were obtained. Du et al. (2018) showed that the *C. krusei* samples were all resistant to floconazole, flucytosine and itraconazole, whereas our study samples were 100% sensitive to phylocytosine. Also, the samples of the *C. parapsylosis* study Du et al. (2018) were resistant to phyllocytosine but sensitive to fluconazole, whereas our samples were 100% sensitive to phylocytosine (20). Noteworthy is the type of population surveyed by Mendes and Du, both studies were conducted on cows’ mastitis, while the present study examined the milk of healthy cows; none of which used medication for treatment.

In a study by Perez et al. (2016) conducted on fungi isolated from birds and ruminants, all of the surveyed *Candida* species were similar to those of the present study, except for *C. glabrata*. They indicated that none of the fungi were resistant to amphotericin B and azole. However, in their research, the type of ruminants was unclear (12).

In the present study, interesting results were obtained by comparing the clinical and cow raw milk isolated non-albicans *Candida* strains. It was found that flucytosine was quite effective on both groups of isolates, and the effect of amphotericin B and fluconazole on *C. parapsylosis* was almost the same in both groups. In the case of *C. krusei*, the results of four antibiotics were more similar to each other, which can be attributed to the inherent resistance of this fungus. Since flucytosine has been less used for livestock; therefore, the sensitivity of non-albicans *Candida* spp. to which was reported as 100% in this study.
